# An efficient approach to finding *Siraitia grosvenorii *triterpene biosynthetic genes by RNA-seq and digital gene expression analysis

**DOI:** 10.1186/1471-2164-12-343

**Published:** 2011-07-05

**Authors:** Qi Tang, Xiaojun Ma, Changming Mo, Iain W Wilson, Cai Song, Huan Zhao, Yanfang Yang, Wei Fu, Deyou Qiu

**Affiliations:** 1 Institute of Medicinal Plant, Chinese Academy of Medical Sciences, Peking Union Medical College, Beijing 100193, China; 2 Guangxi Branch Institute, Institute of Medicinal Plant Development, Chinese Academy of Medical Sciences, Nanning 530023, China; 3 CSIRO Plant Industry, PO Box 1600, Canberra ACT 2001, Australia; 4 The Research Institute of Forestry, Chinese Academy of Forestry, Beijing 100091, China

## Abstract

**Background:**

*Siraitia grosvenorii *(Luohanguo) is an herbaceous perennial plant native to southern China and most prevalent in Guilin city. Its fruit contains a sweet, fleshy, edible pulp that is widely used in traditional Chinese medicine. The major bioactive constituents in the fruit extract are the cucurbitane-type triterpene saponins known as mogrosides. Among them, mogroside V is nearly 300 times sweeter than sucrose. However, little is known about mogrosides biosynthesis in *S. grosvenorii*, especially the late steps of the pathway.

**Results:**

In this study, a cDNA library generated from of equal amount of RNA taken from *S. grosvenorii *fruit at 50 days after flowering (DAF) and 70 DAF were sequenced using Illumina/Solexa platform. More than 48,755,516 high-quality reads from a cDNA library were generated that was assembled into 43,891 unigenes. De novo assembly and gap-filling generated 43,891 unigenes with an average sequence length of 668 base pairs. A total of 26,308 (59.9%) unique sequences were annotated and 11,476 of the unique sequences were assigned to specific metabolic pathways by the Kyoto Encyclopedia of Genes and Genomes. cDNA sequences for all of the known enzymes involved in mogrosides backbone synthesis were identified from our library. Additionally, a total of eighty-five cytochrome P450 (CYP450) and ninety UDP-glucosyltransferase (UDPG) unigenes were identified, some of which appear to encode enzymes responsible for the conversion of the mogroside backbone into the various mogrosides. Digital gene expression profile (DGE) analysis using Solexa sequencing was performed on three important stages of fruit development, and based on their expression pattern, seven *CYP450*s and five *UDPG*s were selected as the candidates most likely to be involved in mogrosides biosynthesis.

**Conclusion:**

A combination of RNA-seq and DGE analysis based on the next generation sequencing technology was shown to be a powerful method for identifying candidate genes encoding enzymes responsible for the biosynthesis of novel secondary metabolites in a non-model plant. Seven *CYP450*s and five *UDPG*s were selected as potential candidates involved in mogrosides biosynthesis. The transcriptome data from this study provides an important resource for understanding the formation of major bioactive constituents in the fruit extract from *S. grosvenorii*.

## Background

*Siraitia grosvenorii*, which belongs to the Cucurbitaceae family, has long been used in traditional Chinese medicine as a natural sweetener and as a folk medicine for the treatment of lung congestion, colds and sore throats. In recent years, important pharmacological characteristics, such as anti-cancer and anti-hyperglycemic effects and inhibition of oxidative modification of low-density lipoprotein, have been reported [1-4]. Many cucurbitane-type triterpene glycosides have been isolated and characterized from the fruits [5-10]. The mixed mogrosides have been estimated to be about 300 times as sweet as sucrose so that an 80% extract was nearly 250 times sweeter than sugar [7]. Among them, mogroside V which accounts to 20% of mogrosides is extremely sweet. The purified, sweet principle, mogroside V, has been approved as a high-intensity sweetening agent in Japan [11] and the non-caloric sweet taste extract is a generally recognized as safe (GRAS) non-nutritive sweetener, flavor enhancer, and food ingredient in the USA [12].

**Figure 1:**
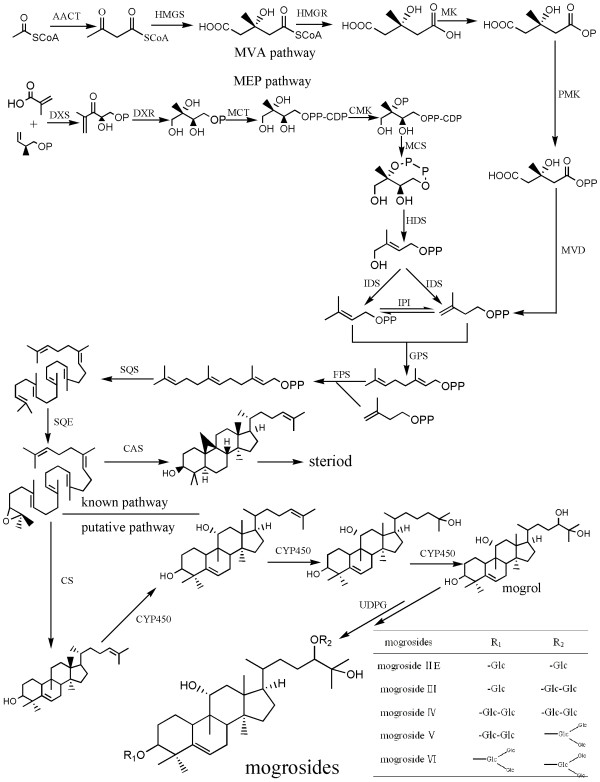
**Putative mogrosides biosynthesis pathway in *Siraitia grosvenorii***. AACT: acetyl-CoA acetyltransferase, EC:2.3.1.9; HMGS: hydroxymethylglutaryl-CoA synthase, EC:2.3.3.10; HMGR: 3-hydroxy-3-methylglutaryl-coenzyme A reductase, EC:1.1.1.34; MK: mevalonate kinase, EC:2.7.1.36; PMK: phosphomevalonate kinase, EC:2.7.4.2; MVD: diphosphomevalonate decarboxylase, EC:4.1.1.33; DXS: 1-deoxy-D-xylulose-5-phosphate synthase, EC:2.2.1.7; DXR: 1-deoxy-D-xylulose-5-phosphate reductoisomerase, EC:1.1.1.267; MCT: 2-C-methyl-D-erythritol 4-phosphate cytidylyltransferase, EC:2.7.7.60; CMK: 4-diphosphocytidyl-2-C-methyl-D-erythritol kinase, EC:2.7.1.148; MCS: 2-C-methyl-D-erythritol 2,4-cyclodiphosphate synthase, EC:4.6.1.12; HDS: 4-hydroxy-3-methylbut-2-enyl diphosphate synthase, EC:1.17.7.1; IDS: 4-hydroxy-3-methylbut-2-enyl diphosphate reductase (isopentenyl/dimethylallyl diphosphate synthase), EC:1.17.1.2; IPI: isopentenyl-diphosphate delta-isomerase, EC:5.3.3.2; GPS: geranyl diphosphate synthase, EC:2.5.1.1; FPS: farnesyl diphosphate synthase/farnesyl pyrophosphate synthetase, EC:2.5.1.10; SQS: squalene synthetase; CAS: cycloartenol synthase, EC:2.5.1.21; SQE: squalene epoxidase, EC:1.14.99.7; CS: cucurbitadienol synthase, EC:5.4.99.8; P450: cytochrome P450, EC:1.14.-.-; and UDPG: UDP-glucosyltransferase, EC:2.4.1.-.

The active components responsible for the sweetness are mogrosides, which are members of the family of triterpene glycosides. In studies the relationships of the structure and the taste, the number of glucose units at the 3 and 24-position of the aglycone moiety are thought responsible for the perception of taste [13]. Among them, mogroside IV, V and mogroside VI which have more glucose units and are extremely sweet, but the fruit also contains some tasteless glycosides, as well as bitter-tasting glycosides such as mogroside III and mogroside II E which possess less glucose units (shown in Figure 1). Previous research has indicated that the bitter mogroside II E and tasteless mogroside III are the main products in the fruit at the early growing stage, but these decrease as the fruit matures [13]. The content of mogroside V increases rapidly from 50 to 70 DAF, and levels off after 85 DAF.

Although the various chemical and pharmacological properties of mogrosides in *S. grosvenorii *have been extensively studied, the biosynthetic pathway of the mogrosides remains poorly understood. Triterpene saponins are synthesized via the isoprenoid pathway by cyclization of 2, 3-oxidosqualene to give primarily cucurbitane skeleton. The triterpenoid backbone then undergoes various modifications (oxidation, substitution and glycosylation) mediated by cytochrome P450-dependent monooxygenases, glycosyltransferases and other enzymes [14]. Mogrosides, cucurbitane-type triterpene glycosides, are synthesized by the isoprenoid pathway and share the same precursor, 2, 3-oxidosqualene, with sterol. In the early stage of active isoprene unit formation, both the cytosolic mevalonate pathway (MVA pathway) and the plastidial 2-*C*methyl-D-erythritol-4-phosphate pathway (MEP pathway) can produce isopentenyl diphosphate (IPP), which is then converted to its allylic isomer, isopentenyl/dimethylallyl diphosphate (DMAPP), through the action of IPP isomerase [15]. Triterpenoids are known to be formed by the MVA pathway because they are cytosolic products. However, there are examples that two pathways can act cooperatively to create a molecule [16]. Little progress has been made toward determining the precise source of isoprene units in mogrosides biosynthesis. Due to the biological importance of sterol and diterpenoid, the previous steps in its conversion from acetyl-CoA (MVA pathway) and 1-deoxy-D-xylulose-5-phosphate (MEP pathway) to IPP have been widely studied in many plant species, but the next steps are still unclear, especially the late steps of the pathway. The cyclization of oxidosqualene is the branch point for the biosynthesis of mogrosides and sterol. However, the portion of the pathway that lies downstream of cyclization remains largely unknown. According to the proposed pathway (shown in Figure 1), some specific CYP450s and UDP-glycosyltransferases (UGTs) may catalyze the conversion of cucurbitadienol to various mogrosides. To date, no genes involved in mogroside biosynthesis have been identified from *S. grosvenorii *or other mogrosides-producing plants.

Next generation sequencing technology (NGS) has emerged as a cost effective approach for high-throughput sequence determination that has dramatically improved the efficiency and speed of gene discovery [17,18]. RNA-Seq refers to whole transcriptome shotgun sequencing wherein mRNA or cDNA is mechanically fragmented, resulting in overlapping short fragments that cover the entire transcriptome. DGE is a tag-based transcriptome sequencing approach where short raw tags are generated by endonuclease. The expression level of genes in the sample is measured by counting the number of individual mRNA molecules produced from each gene. A combination of RNA-Seq and DGE approaches is powerful, as it leverages the advantages of each, enabling both large scale functional assignment of genes via the assembly of large sequenced transcriptome library (RNA-Seq), and the ability to easily perform quantitative gene expression comparisons without potential bias, allowing for a more sensitive and accurate profiling of the transcriptome that more closely resembles the biology of the cell (DGE) [19]. Despite the commercial and medicinal importance of *S. grosvenorii*, RNA-Seq and DGE have not yet been applied to *S. grosvenorii *genomic research.

In this study a cDNA library generated from of equal amount of RNA taken from *S. grosvenorii *fruit at 50 DAF and 70 DAF were sequenced using Illumina/Solexa platform. More than 48,755,516 HQ reads from a cDNA library generated that was assembled into 43,891 unigenes. Bioinformatic analysis indicated that cDNA sequences could be identified that matched all known enzymes involved in the biosynthesis of the mogroside backbone. DGE of *S. grosvenorii *at 3, 50 and 70 DAF identified seven *CYP450*s and five *UDPG*s out of a total of eighty-five *CYP450 *and ninety *UDPG *unigenes, as potential candidate genes responsible for mogroside backbone modifications. These results demonstrated the powerful ability of high-throughput sequencing to identify candidate genes involved in novel metabolic pathways in non-model plant systems.

## Results

### Illumina Sequencing and *de novo *assembly

To obtain an overview of the *S. grosvenorii *fruit transcriptome, a cDNA library was generated from an equal mixture of RNA isolated from 50 DAF and 70 DAF fruits, and pair end sequenced using the Illumina platform. After cleaning and quality checks, 48 million of 75 bp reads were assembled into 98,510 contigs (shown in Table 1). The mean contig size was 313 bp with a length ranging from as small as 75 bp to as large as 7,056 bp. Using paired-end joining and gap-filling, the contigs were further assembled into 67,061 scaffolds with a mean size of 484 bp including 7,929 scaffolds larger than 1,000 bp (Table 1). After clustering using TGICL software [20], the 67,061 scaffolds generated 43,891 unigenes (a scaffold that matches no other scaffold) with a mean size of 668 bp. The size distribution of these contigs, scaffolds and unigenes is shown in Additional file 1.

**Table 1:** Summary for the *Siraitia grosvenorii *transcriptome

Total number of reads	48,755,516
Total base pairs (bp)	3,656,663,700
Average read length	75 bp
Total number of contigs	98,510
Mean length of contigs	323 bp
Total number of scaffolds	67,061
Mean length of scaffolds	484 bp
Total number of unigenes	43,891
Mean length of unigenes	668 bp
Sequences with E-value < 10-5	26,307

### Annotation of predicted proteins

For annotation, distinct gene sequences were first searched using BLASTx against the non-redundant (nr) NCBI nucleotide database using a cut-off E-value of 10-5. Using this approach, 26,307 unigenes (59.9% of all unigenes) returned a significant BLAST result (shown in Additional file 2). Because of the relatively long length of unigenes (mean size of 668 bp) and the addition of 75 Uni-ESTs from a suppression subtractive hybridization cDNA library of *S. grosvernorii *as a reference (data not shown), most of the 43,891 assembled sequences have been matched to known genes (59.9%).

### Gene ontology (GO) classification

**Figure 2:**
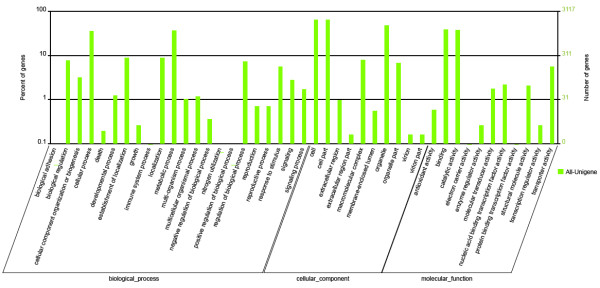
**Histogram of gene ontology classification**. The results are summarized in three main categories: biological process, cellular component and molecular function. The right y-axis indicates the number of genes in a category. The left y-axis indicates the percentage of a specific category of genes in that main category.

GO assignments were used to classify the functions of the predicted *S. grosvenorii *genes. Based on sequence homology, 3,117 sequences can be categorized into 41 functional groups (shown in Figure 2). In each of the three main categories (biological process, cellular component and molecular function) of the GO classification, 'metabolic process', 'cell'&'cell part' and 'Binding' terms are dominant respectively. We also noticed a high-percentage of genes from categories of 'cellular process', 'organelle' and 'catalytic' and only a few genes from terms of 'biological adhesion', 'extracellular region part' and 'nutrient reservoir' (shown in Figure 2). The GO analysis showed that the functions of the identified genes are involved various biological processes. 1,209 sequences were annotated as 'metabolic process' category, which suggests that our study may allow for the identification of novel genes involved in the secondary metabolite synthesis pathways.

### Functional classification by KEGG

Functional classification and pathway assignment was performed by the Kyoto Encyclopedia of Genes and Genomes (KEGG) [21]. First, the 43,891 unique sequences were compared using BLASTX with an E-value cutoff of < 10-5 against the KEGG database. To identify the biological pathways that are active in the *S. grosvenorii*, the 26,307 annotated sequences were mapped to the reference canonical pathways in KEGG. In total, 11,475 sequences were assigned to 217 KEGG pathways. The pathways with most representation by the unigenes were starch and sucrose metabolism (287 members), purine metabolism (287 members) and pyrimidine metabolism (251 members). These annotations provide a valuable resource for investigating specific processes, functions and pathways during *S. grosvenorii *research. Interestingly, 739 unigenes involved in biosynthesis of secondary metabolites were found (shown in Table 2). Among them, the cluster for 'Phenylpropanoid biosynthesis [PATH: ko00940]' represents the largest group (134, 17.4%) followed by 'Limonene and pinene degradation [PATH: ko00903]' (119, 16.1%) and 'Stilbenoid, diarylheptanoid and gingerol biosynthesis [PATH: ko00945]' (85, 11.5%). Mogrosides belong to the terpenoid saponins, which share a common pathway from acetyl-CoA to 2, 3-oxidosqualene with sterol. Most of the enzymes identified in the fruit cDNA library were mapped into the terpenoid backbone [PATH: ko00900] and sterol biosynthesis [PATH: ko00100] groups by KEGG. All of the genes encoding enzymes involved in the biosynthesis of the mogroside backbone were present in our transcriptome of the *S. grosvenorii *fruit (shown in Table 3). In most cases, more than one unigene was annotated as the same enzyme. Such unigenes may represent different fragments of a single transcript, different members of a gene family, or both. Additionally, eighty-five unigenes for *CYP450 *gene and ninety unigenes for *UDPG *were also discovered (shown in Additional file 3). All these unigenes are important resources for *S. grosvenorii *genetic engineering work in future. These results also demonstrated the powerful ability of high-throughput sequencing to identify genes in metabolic pathways.

**Table 2:** The unigenes related to secondary metabolites

Biosynthesis of Secondary Metabolites	Unigene Numbers
Anthocyanin biosynthesis	6
Betalain biosynthesis	4
Brassinosteroid biosynthesis	7
Caffeine metabolism	2
Carotenoid biosynthesis	53
Diterpenoid biosynthesis	37
Flavone and flavonol biosynthesis	11
Flavonoid biosynthesis	34
Glucosinolate biosynthesis	7
Indole alkaloid biosynthesis	27
Isoquinoline alkaloid biosynthesis	29
Limonene and pinene degradation	119
Novobiocin biosynthesis	24
Phenylpropanoid biosynthesis	134
Stilbenoid, diarylheptanoid and gingerol biosynthesis	85
Streptomycin biosynthesis	37
Terpenoid backbone biosynthesis	63
Tetracycline biosynthesis	12
Tropane, piperidine and pyridine alkaloid biosynthesis	22
Zeatin biosynthesis	26

Total	739

**Table 3:** The numbers of Unigene involved in mogrosides biosynthesis

Gene	**Enzyme No**.	Numbers
1-deoxy-D-xylulose-5-phosphate synthase (DXS)	[EC:2.2.1.7]	11
1-deoxy-D-xylulose-5-phosphate reductoisomerase (DXR)	[EC:1.1.1.267]	4
2-C-methyl-D-erythritol 4-phosphate cytidylyltransferase (MCT)	[EC:2.7.7.60]	1
4-diphosphocytidyl-2-C-methyl-D-erythritol kinase (CMK)	[EC:2.7.1.148]	2
2-C-methyl-D-erythritol 2,4-cyclodiphosphate synthase (MCS)	[EC:4.6.1.12]	2
4-hydroxy-3-methylbut-2-enyl diphosphate synthase (HDS)	[EC:1.17.7.1]	1
4-hydroxy-3-methylbut-2-enyl diphosphate reductase (HDR/IDS)	[EC:1.17.1.2]	1
acetyl-CoA acetyltransferase (AACT)	[EC:2.3.1.9]	5
hydroxymethylglutaryl-CoA synthase (HMGS)	[EC:2.3.3.10]	1
3-hydroxy-3-methylglutaryl-coenzyme A reductase (HMGR)	[EC:1.1.1.34]	3
mevalonate kinase (MK)	[EC:2.7.1.36]	4
phosphomevalonate kinase (PMK)	[EC:2.7.4.2]	3
diphosphomevalonate decarboxylase (MVD)	[EC:4.1.1.33]	2
isopentenyl-diphosphate delta-isomerase (IPI)	[EC:5.3.3.2]	5
geranyl diphosphate synthase (GPS)	[EC:2.5.1.1]	1
farnesyl diphosphate synthase (FPS)	[EC:2.5.1.10]	2
hexaprenyl pyrophosphate synthetase (HPS)	[EC:2.5.1.33]	1
geranylgeranyl pyrophosphate synthetase (GGPS)	[EC:2.5.1.29]	1
squalene synthetase (SQS)	[EC:2.5.1.21]	3
squalene epoxidase (SQE)	[EC:1.14.99.7]	1
cycloartenol synthase (CAS)	[EC:5.4.99.8]	5
cucurbitadienol synthase (CS)		1

Total		60

### Changes in gene expression profiles during different developmental stages

DGE generates absolute rather than relative gene expression measurements and avoids many of the inherent limitations of microarray analysis. DGE was used to analyze the gene expression during three stages of *S. grovenorii *fruit development. Three DGE libraries: 3, 50 and 70 DAF, were sequenced that generated between 3.4 to 3.7 million high quality tags and the number of tag entities with unique nucleotide sequences ranged from 85,737 to 91,130 (shown in Table 4). The distributions of total tags and distinct tags over different tag abundance categories are shown in Additional file 4. The tag sequences were mapped to *S. grosvenorii *reference transcriptome database. Between 46,700 to 51,320 distinct tags were mapped to a gene in the reference database and up to 49.58% (21,761) of the sequences in transcriptome reference tag database could be unequivocally identified by unique tag (shown in Table 4).

**Table 4:** Statistics of DGE sequencing

Summary		3 DAF	50 DAF	70 DAF
Raw Data	Total	3510563	3738193	3784929
Raw Data	Distinct Tag	207988	202720	184923
Clean Tag	Total number	3382707	3612715	3682563
Clean Tag	Distinct Tag number	87140	85737	91130
All Tag Mapping to Gene	Total number	1573645	1237119	1379598
All Tag Mapping to Gene	Total % of clean tag	46.52%	34.24%	37.46%
All Tag Mapping to Gene	Distinct Tag number	46700	46865	51320
All Tag Mapping to Gene	Distinct Tag % of clean tag	53.59%	54.66%	56.32%
Unambiguous Tag Mapping to Gene	Total number	1566698	1233379	1376138
Unambiguous Tag Mapping to Gene	Total % of clean tag	46.31%	34.14%	37.37%
Unambiguous Tag Mapping to Gene	Distinct Tag number	46520	46689	51138
Unambiguous Tag Mapping to Gene	Distinct Tag % of clean tag	53.39%	54.46%	56.12%
All Tag-mapped Genes	number	19607	21534	21865
All Tag-mapped Genes	% of ref genes	44.67%	49.06%	49.82%
Unambiguous Tag-mapped Genes	number	19492	21406	21761
Unambiguous Tag-mapped Genes	% of ref genes	44.41%	48.77%	49.58%
Unknown Tag	Total number	1809062	2375596	2302965
Unknown Tag	Total % of clean tag	53.48%	65.76%	62.54%
Unknown Tag	Distinct Tag number	40440	38872	39810
Unknown Tag	Distinct Tag % of clean tag	46.41%	45.34%	43.68%

**Figure 3:**
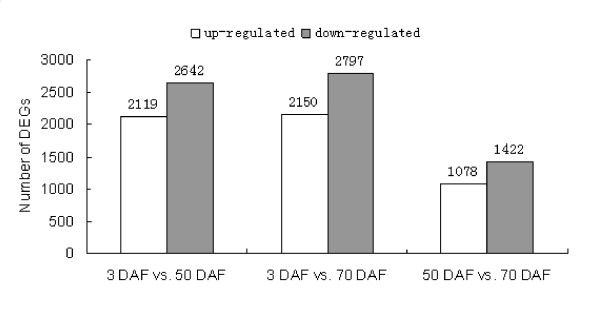
**Changes in gene expression profile among the different developmental stages**. The number of up-regulated and down-regulated genes between 3 DAF and 50 DAF; 3 DAF and 70 DAF; 50 DAF and 70 DAF are summarized. DEGs: Differentially Expressed Genes.

Differentially expressed tags between samples were identified by an algorithm developed by Audic *et al *[22]. As expected the majority of gene expression changes occurred between the DAF 3 and DAF 50/DAF 70 (Figure 3 & Additional file 5, 6, 7) with slightly more down-regulated genes observed. More than 40%-45% of the highly regulated genes were found to be orphan sequences - no homologues found in the NCBI database. This may indicate that *S. grosvenorii *fruit development contains many unique processes and pathways.

### Expression levels of putative genes involved in mogrosides biosynthesis

**Figure 4:**
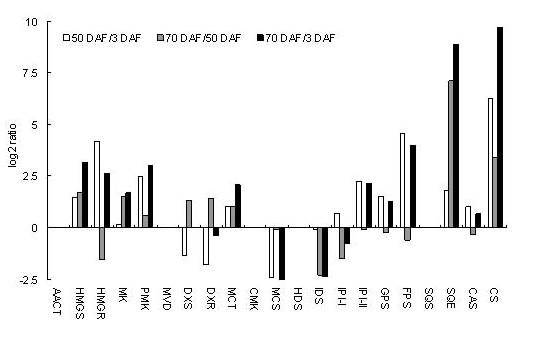
**Expression pattern of genes involved in mogrosides biosynthesis at different fruit developmental stages by DGE**. 21 genes expression in 3 DAF, 50 DAF and 70 DAF were analyzed by DGE.

As shown in Figure 4, to examine the expression of the 21 genes putatively involved in mogrosides backbone biosynthesis revealed very low expression levels of *AACT*, *MVD*, *CMK*, *HDS*, *SQS *genes. The 16 remaining genes had significant expression levels that showed changes in gene expression during fruit development (shown in Figure 4). Between 3 DAF and 50 DAF, *DXS*, *DXR*, *MCS*, *IDS *are down-regulated, whereas the remaining 12 genes are up-regulated. Between 50 DAF and 70 DAF, *HMGR*, *IDS*, *IPI*-I, *IPI*-II, *GPS*, *FPS *and *CAS *were found to be down-regulated, whereas, the other 9 genes were up-regulated. Overall the trend between 3 DAF and 70 DAF was that *DXR*, *MCS*, *IDS*, *IPI *-I showed decreased expression over time, while *HMGS*, *HMGR*, *MK*, *PMK*, *MCT*, *IPI*-II, *GPS*, *FPS*, *SQS*, *CAS*, and *CS *genes showed degrees of up-regulation. *SQE *and *CS *genes were found to change the most in this period, (8.9 times and 9.7 times respectively). The rapid accumulation of mogroside V from 50 DAF to 70 DAF may result from the increase in expression of *SQE *and *CS*. *SQE *plays a key role in the cyclical transformation of squalene to 2, 3-oxidosqualene. The cyclization of 2, 3-oxidosqualene is the branch point of sterol and mogrosides biosynthesis and controls the carbon flux through the branched biosynthetic pathways. The next important step in the pathway is via *CS *and *CAS*, which catalysis 2, 3-oxidosqualene formed cucurbitadenol and sterol respectively [23]. Cucurbitadenol is the precursor of different mogrosides. To date, no genes involved in mogroside biosynthesis have been cloned from *S. grosvenorii *or other mogrosides-producing plants. The correlation between the expression of *SQE *and *CS *and the rapid formation of mogrol during fruit development presents a way of finding possible *CYP450 *and *UDPG *genes involved in mogrosides biosynthesis via identification of candidate genes with similar expression profiles. This method of finding candidate genes has successfully identified a gene involved in saponin biosynthesis [24].

### Candidate CYP450 enzymes involved in mogrosides biosynthesis

**Figure 5:**
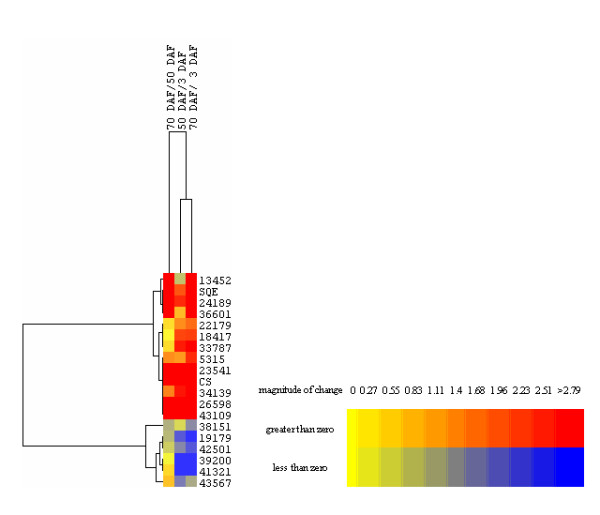
**Clustering of CYP450 genes expression profiles at three different fruit developmental stages**. Hierarchical clustering of expression data for 17 candidate CYP450 genes using CS and SQE as reference profiles. Expression ratios are expressed as Log 2 values.

CYP450 proteins are the largest family of plant proteins and catalyze most of the oxidation steps in plant secondary metabolism [25,26]. In the biosynthetic pathway of mogrosides, the conversion from cucurbitadenol to mogrol is catalyzed by one or more CYP450s. A total of eighty-five unigenes were annotated as *CYP450*s in our *S. grosvenorii *transcriptome (shown in Additional file 8). To find the *CYP450*s putatively involved in mogrosides biosynthesis, these unigenes expression profiles were compared to *SQE *and *CS *by DGE (shown in Figure 5). From 3 DAF to 70 DAF, the *SQE *and *CS *genes are up-regulated, and so only *CYP450*s that displayed some up-regulation during this developmental period were considered candidates for mogrosides biosynthesis. A total of seventeen *CYP450*s that were found to be differentially expressed between at least one of the three developmental time points were chosen, and their expression profiles hierarchically clustered with that of *SQE *and *CS *(shown in Figure 5). Seven candidate *CYP450*s were found to cluster closely with either *SQE *or *CS *(correlation > 0.99): Unigene5315 (transcriptome short reads assembly (TSA) accession number JL554661), Unigene23541 (JL554662), Unigene24189 (JL554663), Unigene26598 (JL554664), Unigene34139 (JL554665), Unigene36601 (JL554666) and Unigene43109 (JL554667). These genes are therefore promising candidates that may catalyze the oxidation of cucurbitadinol and the formation of mogrol.

### Candidate UDPG enzymes involved in mogrosides biosynthesis

**Figure 6:**
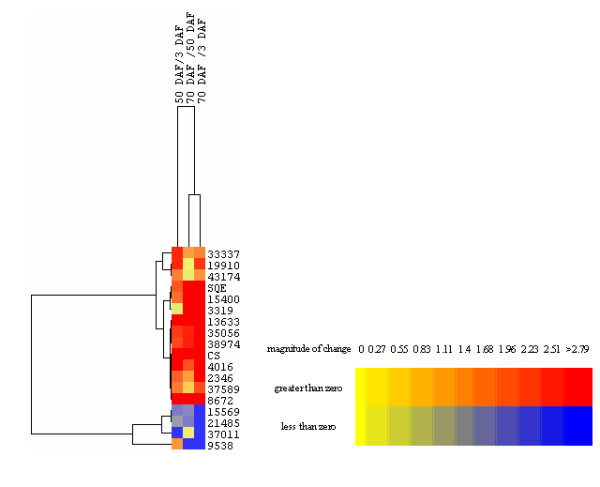
**Clustering of UDPG genes expression profiles at three different fruit developmental stages**. Hierarchical clustering of expression data for 16 candidate *UDPG *genes using CS and SQE as reference profiles. Expression ratios are expressed as Log 2 values.

Glucosyltransferases (UDPG) are another large multi-gene family in plants. In general, glycosylation is the last step in the biosynthesis of secondary metabolites and sugar conjugation results in both increased stability and water solubility [27-29]. The *S. grosovenorii *cDNA library contained seventy-two glycosyltransferase and ninety glucosyltransferase unigenes (shown in Additional file 9). Only glucosyltransferases not glycosyltransferases, which used glucose as the donor and mogrol as the acceptor, catalyzed the formation of mogrosides. Similar to the candidate *CYP450*s, 16 differentially expressed *UDPG*s that showed up-regulation of gene expression during fruit development were selected and their expression profiles hierarchically clustered with that of *SQE *and *CS *(shown in Figure 6). The expression pattern of five *UDPG*s showed strong correlation to that of *SQE *and *CS *(> 0.99, Figure 6) and appear the best *UDPG*s candidates for encoding the enzymes responsible for mogrosiedes biosynthesis. These *UDPG*s consisted of Unigene2346 (JL554668), Unigene4016 (JL554669), Unigene8672 (JL554670), Unigene15400 (JL554671), and Unigene37589 (JL554672).

## Discussion

EST analysis is one of the most valuable tools for gene discovery. However, until the advent of NGS, significant coverage of the transcriptome was labor intensive and expensive. Therefore, EST analyses tended to identified only a few candidate genes involved in complex biosynthetic pathways such as that observed from studies in *Panax quinquefolius *and *Panax ginseng *[30-32]. Even in combination with subtracted cDNA libraries strategies in *S. grosvenorii*, traditional EST sequencing only identified one *CYP450 *and two *Glycosyltransferase*s (1, 200 ESTs) and none potentially involved in mogrosides biosynthesis (data not shown). The development of NGS has removed these limitations, and a number of successful identifications of secondary metabolism genes from plants with little genomics sequence information including cotton [33], *American ginseng *[34], *Glycyrrhiza uralensis *[35], *Medicago truncatula *[24,36], *Huperzia serrata *and *Phlegmariurus carinatus *[37] have been performed.

Despite the commercial and medicinal importance of *S. grosvenorii*, molecular information on this species is lacking preventing analysis of the mogrosides biosysnthetic pathway. Creating a cDNA library from the *S. grosvenorii *fruit and performing RNA-seq and DGE provided a very efficient means for identifying the genes associated with known enzymes involved in the biosynthesis of secondary metabolites and for providing candidate genes that could be associated with currently unknown steps in the pathway. Thus, candidates for all of the known genes encoding enzymes involved in mogrosides backbone biosynthesis, including two 2, 3-oxidosqualene cyclases: *CAS *and *CS *were identified in this study. Very little is known about the late stages of mogrosides biosynthesis. This part of the pathway includes multiple oxidation and glycosylation steps catalyzed by enzymes from the CYP450 and glucosyltransferase superfamilies, respectively. These families of enzymes display a wide range of substrate specificities and are responsible for the diversity of many plant secondary metabolites. For example, approximately one hundred and twenty *UGT *genes and two hundreds and seventy-two *CYP450 *genes were identified in the model plant *Arabidopsis thaliana*. Because of the biological, pharmacological, and agricultural importance of secondary metabolites, *UGT*s and *CYP450*s have attracted considerable interest for decades, but only a few have been characterized by traditional biochemistry and genetics. To date, only two CYP450s involved in triterpene saponin biosynthesis are functionally characterized: a β-amyrin 11-oxidase from *Glycyrrhiza uralensis *[38] and a β-amyrin and sophoradiol 24-hydroxylase from *Glycine max *[39]. Also, five UGTs in triterpene saponin biosynthesis have been identified: soybean [40], UGT73K1, UGT71G1 and UGT73F3 from *Medicago truncatula *[24,41] and UGT74M1 from *Saponaria vaccaria *[42]. Genomic and co-expression analyses by DNA microarray provide a basis for future studies to define genetically the roles of multiple cytochromes P450 and glycosyltransferases in triterpene saponin biosynthesis in model plant *Medicago truncatula*. UGT73F3 have been identified for glucosylating hederagenin at the C28 position [24]. However, all of the aforementioned enzymes are involved in soyasapogenol and oleanane-type ginsenoside biosynthesis. No *CYP450*s or *UGT*s in the cucurbitane-type mogrosides biosynthetic pathway have been previously functionally characterized. Therefore, this study focused on the discovery of *CYP450*s and *UGT*s involved in the biosynthesis of cucurbitane-type mogrosides, which are the major mogrosides type in the *S. grosvenorii *fruit.

In the cucurbitane-type mogorisdes biosynthetic pathway, CYP450s catalyze the C6 and C12 hydroxylation of cucurbitadienol, while glycosylation generally occurs on C3 and C24 of the aglycones. In total, eighty-five *CYP450 *unigenes and ninety *UDPG *unigenes were found in the *S. grosvenorii *cDNA library. However, the large number of candidate genes produced by NGS leads to difficulty in the characterization of the enzymes that are actually involved in this pathway. One CYP450 and four UDPG were selected as the candidates most likely to be involved in ginsenoside biosynthesis through a methyl jasmonate (MeJA) inducibility experiment and tissue-specific expression pattern analysis based on NGS and real-time PCR assay [34]. Three CYP450 enzymes and six UGTS were selected as the candidates most likely to be involved in glycyrrhizin biosynthesis by the same approach [35]. The real-time PCR method is time-consuming and labor intensive, as it can only study a few genes at the same time. DNA microarrays are mainly used in model plants as they generally require either extensive gene sequence information or large scale amplification of cDNA libraries. Unfortunately, there is little information about gene cloning and EST in NCBI database in *S. grosvenorii*. In contrast, the DGE method based on high-throughput sequencing technology in combination with NGS transcriptome information, is more suitable and affordable for tens of thousands gene expressions studies at once. Therefore, in this study, the DGE of three different development stages were carried out to try to select those *CYP450*s and *UGT*s most likely to be involved in mogrosides biosynthesis. The expression levels of 20 genes involved in mogrosides backbone biosynthesis all showed some variation in expression pattern through DGE analysis. Among these, *SQE *and *CS *genes in late steps changed the most. They increased 7.11 times and 3.40 times respectively from 50 DAF to 70 DAF, increased 8.89 times and 9.69 times respectively in 70 DAF vs. 3 DAF. Mogroside V is the main constituent of ripe fruits, while mogroside III and II E are the leading components in unripe fruits [13]. At the early growing stage (from 3 DAF to 50 DAF), mogroside V increases from 0.057% to 0.083%, then increases rapidly to 2.150% (25.9 times) from 50 DAF to 70 DAF and gets stability at 2.700% after 85 DAF. Therefore levels of *SQE *and *CS *gene expression levels are coincident with mogroside V content. Selecting *SQE *and *CS *as reference expression patterns for *CYP450*s and *UDPG*s that would be functional at the same development periods, similar to the work on saponin biosynthesis [24], identified only seven *CYP450*s and five *UDPG*s candidates. The function of these candidate *CYP450*s and *UDPG*s will be confirmed by heterologous expression in *Escherichia coli *or yeast, and *in vitro *enzymatic assay.

## Conclusion

This study demonstrates the feasibility of using combination of RNA-Seq and DGE to provide a method for identifying and studying the genes involved in secondary metabolism not only in *S. grosvenorii *but also other non-model medicinal plants. Candidate genes encoding enzymes potentially involved in mogrosides backbone biosynthesis as well as *CYP450 *and *UDPG *genes likely to be involved in mogrosides biosynthesis were rapidly identified by this approach.

## Methods

### Sample collection and preparation

Routinely, the *S. grosvenorii *fruits are harvested between 70 DAF to 85 DAF for medicinal use. The mogroside V appears in 50 DAF and peaks after 70 DAF. Therefore, 50 DAF and 70 DAF *S. grosvenorii *were collected for transcriptome sequencing from the field in Xing-an County, Guilin, Guangxi autonomous region, China. 3 DAF, 50 DAF and 70 DAF fruits were using for DGE analysis. The plant tissues were then cut into small pieces and were immediately frozen in liquid nitrogen. All materials were stored at -80°C until further processing.

### RNA isolation and library preparation for transcriptome analysis

Total RNA was isolated using the Trizol reagent (Invitrogen). RNA samples were treated with Dnase I (Promega) at a concentration of 1 unit/μg of total RNA. Total RNA purity and degradation were checked on 1% agarose gels before proceeding. The samples for transcriptome analysis were prepared using the Illumina's kit following the manufacturer's recommendations. Briefly, mRNA was purified from 6 μg of total RNA using oligo (dT) magnetic beads. Following purification, the mRNA was fragmented into small pieces using divalent cations under elevated temperature and the cleaved RNA fragments were used for first strand cDNA synthesis using reverse transcriptase and random primers. This was followed by second strand cDNA synthesis using DNA polymerase I and RNaseH. These cDNA fragments then went through an end repair process and ligation of adapters. These products were purified and enriched with PCR to create the final cDNA library.

### Analysis of Illumina sequencing results

The cDNA library was sequenced on the Illumina sequencing platform (GAII). The size of the library is approximately 200 bp and both ends of the libraries are sequenced. Sequences from the Illumia sequencing were deposited in the GenBank Short Read Archive (Accession SRP006841). Image deconvolution and quality value calculations were performed using the Illumina GA pipeline 1.3. The raw reads were cleaned by removing adaptor sequences, empty reads and low quality sequences (reads with unknown sequences 'N'). The reads obtained were randomly clipped into 21 bp K-mers for assembly using de Bruijn graph and SOAP denovo software [43]. After assessing different K-mer sizes, we found that the 21-mer provided the best result for transcriptome assembly. Small K-mers make the graph very complex; while large K-mers can have poor overlap in regions with low sequencing depth. After sequence assembly, the resultant contigs were joined into scaffolds using the read mate pairs. To obtained distinct gene sequences, the scaffolds were clustered using TGI Clustering tools [44]. Distinct sequences were used for blast search and annotation against an NCBI nr database using an E-value cut-off of 10-5. Functional annotation by gene ontology terms (GO, http://www.geneontology.org) was analyzed by Blast2go software. The KEGG pathways annotation was performed using Blastall software against the KEGG database. The assembled sequences can be searched using the Gene-ID listed in Additional file 3.

### DGE library preparation and sequencing

Tag library preparation for the three different growth period samples (3 DAF, 50 DAF, and 70 DAF) was performed in parallel using the Illumina gene expression sample preparation kit. Briefly, total RNA from the three samples was used for mRNA capture with magnetic oligo (dT) beads. First and second strand cDNA were synthesized and beadbound cDNA was subsequently digested with NlaIII. The cDNA fragments with 3' ends were then purified with magnetic beads and Illumina adapter 1 was added to their 5' ends. The junction of the Illumina adapter 1 and CATG site is the recognition site of MmeI, which cuts 17 bp downstream of the CATG site, producing tags with adapter 1. After removing 3' fragments with magnetic beads precipitation, Illumina adapter 2 was introduced at 3' ends of tags, acquiring tags with different adapters at both ends to form a tag library. After 15 cycles of linear PCR amplification, 85 base strips were purified by PAGE gel electrophoresis. These strips were then digested, and the single-chain molecules were fixed onto the Illumina sequencing chip for sequencing. The reproducibility of DGE were > 0.99 [45]. The sequences from the DGE analysis were deposited in the GenBank Short Read Archive (Accession SRP006090).

### Analysis and mapping of DGE tags

Sequencing-received raw image data was transformed by base calling into sequence data. Prior to mapping reads to the reference database, we filtered all sequences to remove adaptor sequence, low quality sequences (tags with unknown sequences 'N'), empty tags (sequence with only adaptor sequences but no tags); low complexity, and tags with a copy number of 1 (probably sequencing error). A preprocessed database of all possible CATG+17 nucleotide tag sequences was created using our transcriptome reference database. For annotation, all tags were mapped to the reference sequences and only allowed 1 or fewer nucleotide mismatches. All the tags mapped to reference sequences from multiple genes were filtered and the remaining tags were designed as unambiguous tags. For gene expression analysis, the number of expressed tags was calculated and then normalized to TPM (number of transcripts per million tags); and the differentially expressed tags were used for mapping and annotation. The complete lists of differentially expressed genes are shown in Additional file 5, 6 and 7.

### Evaluation of DGE libraries

A statistical analysis of the frequency of each tag in the different cDNA libraries was performed to compare gene-expression in different developmental stages. Statistical comparison was performed using the method described by Audic *et al *[22]. FDR (false discovery rate) was used to determine the threshold of P-value in multiple test and analysis. We used FDR < 0.001 as the threshold to judge the significance of gene expression difference. For pathway enrichment analysis, we mapped all differentially expressed genes to terms in the KEGG database and looked for significantly enriched KEGG terms compared to the genome background.

### Clustering of *CYP450 *and *UDPG *gene expression profiles

Hierarchical clustering of log-transformed expression data was carried out using the Cluster 3.0 and Treeview programs [46]. Correlations between gene clusters were determined using Pearson's correlation. Heat maps were constructed using the University of Toronto BAR Heatmapper tool http://www.bar.utoronto.ca/ntools/cgi-bin/ntools_heatmapper.cgi.

## List of abbreviations

DGE: Digital Gene Expression; DAF: days after flowering; MVA: mevalonate pathway; MEP: 2-*C*methyl-D-erythritol-4-phosphate pathway; IPP: isopentenyl diphosphate; DMAPP: isopentenyl/dimethylallyl diphosphate; NGS: Next generation sequencing technology; GO: Gene Ontology; KEGG: Kyoto Encyclopedia of Genes and Genomes; AACT: acetyl-CoA acetyltransferase; HMGS: hydroxymethylglutaryl-CoA synthase; HMGR: 3-hydroxy-3-methylglutaryl-coenzyme A reductase; MK: mevalonate kinase; PMK: phosphomevalonate kinase; MVD: diphosphomevalonate decarboxylase; DXS: 1-deoxy-D-xylulose-5-phosphate synthase; DXR: 1-deoxy-D-xylulose-5-phosphate reductoisomerase; MCT: 2-C-methyl-D-erythritol 4-phosphate cytidylyltransferase; CMK: 4-diphosphocytidyl-2-C-methyl-D-erythritol kinase; MCS: 2-C-methyl-D-erythritol 2,4-cyclodiphosphate synthase; HDS: 4-hydroxy-3-methylbut-2-enyl diphosphate synthase; IDS: 4-hydroxy-3-methylbut-2-enyl diphosphate reductase (isopentenyl/dimethylallyl diphosphate synthase); IPI: isopentenyl-diphosphate delta-isomerase; GPS: geranyl diphosphate synthase; FPS: farnesyl diphosphate synthase/farnesyl pyrophosphate synthetase; SQS: squalene synthetase; SQE: squalene epoxidase; CAS: cycloartenol synthase; CS: cucurbitadienol synthase; CYP450: cytochrome P450; UDPG: UDP-glucosyltransferase.

## Authors' contributions

QT conceived the study, designed and built the cDNA library, participated in data analysis, and drafted the manuscript. XJM initiated the project, helped to conceive the study, and participated in the design and coordination. DYQ designed the study and data analysis. IW participated in clustering of gene expression profiles. CS participated in manuscript formatting and editing. CMM participated in experiment materials preparation. YFY, WF and HZ participated in RNA extraction and RACE experiments. All authors read and approved the final manuscript.

## Supplementary Material

Additional file 1 **Overview of Siraitia grosvenorii transcriptome sequencing and assembly**. (A) Size distribution of Illumina sequencing contigs. (B) Size distribution of Illumina sequencing scaffolds and which after paired-end and gap filling. (C) Size distribution of Illumina sequencing unigenes and which after paired-end and gap filling. Click here for file

Additional file 2 **Top BLAST hits from NCBI nr database**. BLAST results against the NCBI nr database for all the distinct sequences with a cut-off E value above 10-5 are shown. Click here for file

Additional file 3 **Top BLAST hits from NCBI Swissprot database**. BLAST results against the NCBI Swissprot database for all the distinct sequences with a cut-off E value above 10-5 are shown. Click here for file

Additional file 4 **Distribution of total tags and distinct tags over different tag abundance categories**. (A) Distribution of total clean tags. Numbers in the square brackets indicate the range of copy numbers for a specific category of tags. For example, [2,5] means all the tags in this category has 2 to 5 copies. Numbers in the parentheses show the total tag copy number and ratio for all the tags in that category. (B) Distribution of distinct clean tags. Numbers in the square brackets indicate the range of copy numbers for a specific category of tags. Numbers in the parentheses show the total types of tags in that category. Click here for file

Additional file 5 **Differentially expressed genes between 3 DAF and 50 DAF**. TPM: transcript copies per million tags. Raw intensity: the total number of tags sequenced for each gene. FDR: false discovery rate. We used FDR < 0.001 and the absolute value of log2Ratio ≤ 1 as the threshold to judge the significance of gene expression difference. In order to calculate the log2Ratio and FDR, we used TPM value of 0.001 instead of 0 for genes that do not express in one sample. Click here for file

Additional file 6 Differentially expressed genes between 3 DAF and 70 DAF Click here for file

Additional file 7 Differentially expressed genes between 50 DAF and 70 DAF Click here for file

Additional file 8 Putative *CYP450 *genes in the Solexa cDNA library Click here for file

Additional file 9 Putative *UDPG *genes in the Solexa cDNA library Click here for file
