# Supplementary figures and images for: An efficient approach to finding Siraitia grosvenorii triterpene biosynthetic genes by RNA-seq and digital gene expression analysis
Source: BMC Genomics. 2011 Jul 5;12:343. doi: 10.1186/1471-2164-12-343 (PMC3161973; doi:10.1186/1471-2164-12-343)

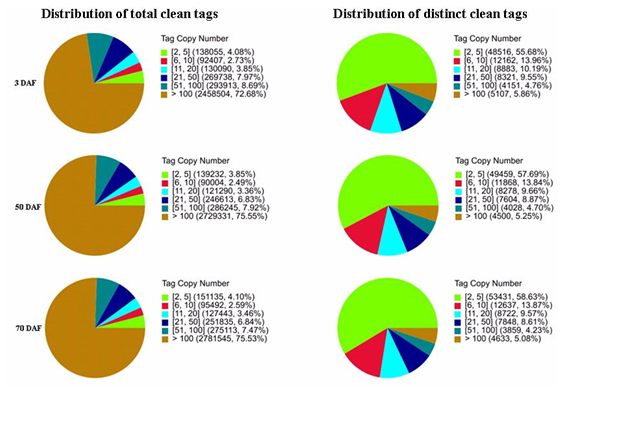

Supplement: Additional file 4 — Distribution of total tags and distinct tags over different tag abundance categories. (A) Distribution of total clean tags. Numbers in the square brackets indicate the range of copy numbers for a specific category of tags. For example, [2,5] means all the tags in this category has 2 to 5 copies. Numbers in the parentheses show the total tag copy number and ratio for all the tags in that category. (B) Distribution of distinct clean tags. Numbers in the square brackets indicate the range of copy numbers for a specific category of tags. Numbers in the parentheses show the total types of tags in that category. [file 1471-2164-12-343-S4.TIFF]
